# Hippocampal neuroplasticity, major depression and, not to forget: ECT

**DOI:** 10.1038/s41380-022-01746-w

**Published:** 2022-08-29

**Authors:** Alexander Sartorius, Sebastian Karl, David Zilles-Wegner

**Affiliations:** 1 Department of Psychiatry and Psychotherapy, Central Institute of Mental Health, Medical Faculty Mannheim, Heidelberg University, J5, 68159 Mannheim, Germany; 2 Department of Psychiatry and Psychotherapy, University Medical Center Göttingen, von-Siebold-Str. 5, 37075 Göttingen, Germany

**Keywords:** Depression, Diagnostic markers

## To the Editor:

With this letter we would like to refer to the review on the topic of neuroplasticity, hippocampus and depression, in which, however, electroconvulsive therapy (ECT) was overlooked [1]. In our opinion, no other specific form of psychiatric therapy plays a more important role for the neuroplasticity hypothesis of depression than ECT, which we would like to highlight by the following.

The neurotrophin/neuroplasticity hypothesis has historically evolved from the catecholamine hypothesis [2], which posits that that depletion of monoamines such as serotonin or norepinephrine can trigger depression. Normalization of the concentration of monoamines in the synaptic cleft (e.g., by selective serotonin reuptake inhibitors) does not immediately lead to remission of depressive symptoms, which suggests delayed changes on the level of gene activation [3]. This finding led to the suggestion – almost 20 years ago – that development of new medications might focus more on downstream changes [4], where ECT was already prominently featured. Tartt et al. mention that the delayed action of selective serotonin and serotonin norepinephrine reuptake inhibitors increased the need for rapid acting antidepressants – ECT is exactly that, and likely because it directly induces downstream changes [5, 6].

## Findings from animal models

Since the authors describe magnetic resonance spectroscopy (MRS) in the context of measuring GABA and glutamate concentrations [1], it seems noteworthy that in an animal model of depression, electroconvulsive shock (ECS, the analogue to ECT) led to a normalization of altered glutamate/GABA ratios within the prefrontal cortex (PFC) and hippocampus [7].

ECS also leads to a dose-dependent increase of hippocampal dendritic arborization and dose-dependent cell proliferation in the subgranular region [8–10]. Further, ECS series and “maintenance” ECS induced a significant increase in newborn neurons in mice hippocampi, suggesting a cellular mechanism for the beneficial effect of ECT [10, 11]. This finding was replicated [12] and extended for synaptogenesis indicating that neuronal survival is key to the efficacy of ECS [13]. ECS elevates hippocampal cell proliferation, while repetitive transcranial magnetic stimulation (rTMS) does not [14].

For brain-derived neurotrophic factor (BDNF), ECS induces a tissue concentration increase in hippocampus and PFC while concentration of BDNF in peripheral serum takes longer (days) to come to a new equilibrium [15].

## Patient findings

BDNF is lower in depressed patients’ serum and rises with ECT, both of which is supported by meta-analytical findings [16, 17]. Additionally, like in the animal models, there is evidence that peripheral BDNF concentrations reach a new equilibrium with some delay after ECT [18]. Consequently, researchers looked for ECT induced hippocampal grey matter volume increases, which were first described by a Swedish group [19] and have since been replicated in large multisite samples [20, 21]. While a mega-analysis did not find a positive association of hippocampal volume change and clinical outcome, a more recent smaller study did find larger hippocampal volume increases in ECT remitters vs. non-remitters [22].

Initial genetic findings corroborate an influence of ECT on e.g. DNA methylation: RAP-GEF2, a protein-encoding gene suggested to be involved in signal transmission and in BDNF receptor pathway signaling in depression is associated with ECT as well as FKBP5, a gene that is involved in stress hormone regulation [23].

MRS-studies in depressed patients treated with ECT also showed a normalization of glutamate levels in the hippocampus and anterior cingulate cortex which were associated with both ECT and symptom improvement [24, 25].

Another relevant aspect mentioned in the introduction of Tartt et al. [1] concerns the assumed relationship between immunologic and neurotrophic processes in the hippocampus. Regarding ECT, decreased systemic levels of interleukins and cortisol after an ECT series have been described in a systematic review [26] and increased immune activation measured in the cerebrospinal fluid at baseline has been shown to predict better seizure quality [27] and better treatment response to ECT [28]. Given the inverse relationship between cortisol exposition and hippocampal volumes in both animal and human studies [29], the reduction of inflammatory processes by ECT may also contribute to increased hippocampal volumes after ECT [30] in addition to more direct neurotrophic effects. There is some evidence from ECT research that inflammatory activity can influence the relationship between BDNF and ECT treatment outcomes [31]. However, it may be that neuroplastic effects of ECT are necessary but not sufficient for a response. Other hypotheses include that ECT-induced seizures elicit a variety of processes in the brain, some of which having antidepressant effects, others having anticatatonic effects, and yet others leading to a grey matter increase [32, 33].

To conclude, ECT research has had important influence on the development of the hippocampal neurotrophin/neuroplasticity hypothesis (and other hypotheses) of depression [33], which should not be overlooked. With the inclusion of severely ill patients and a large antidepressant effect size, ECT studies in particular offer optimal conditions to make contributions to the elucidation of the etiopathogenesis of depression.
